# First Assessment of Genetic Damage in the Speckled Cockroach (*Nauphoeta cinerea*) After Consumption of Lettuce (*Lactuca sativa*) Cultivated and Commercialized in Northeastern, Brazil

**DOI:** 10.1002/tox.70020

**Published:** 2026-01-05

**Authors:** Aleson Aparecido da Silva, Eduardo Henrique da Silva Melo, Érima Maria de Amorim, Maria Gislaine Pereira, Claudia Rohde

**Affiliations:** ^1^ Programa de Pós‐Graduação Em Biologia Celular e Molecular Aplicada Universidade de Pernambuco Recife Pernambuco Brazil; ^2^ Centro Acadêmico de Vitória Universidade Federal de Pernambuco Vitória de Santo Antão Pernambuco Brazil

**Keywords:** bioindicator, crops, environmental contamination, genotoxicity, insect

## Abstract

This study reports, for the first time, the use of the speckled cockroach *Nauphoeta cinerea* as a promising bioindicator for genotoxic monitoring. It was validated through control groups (both positive and negative) in addition to testing lettuce ( *Lactuca sativa* ) samples from public street markets consumed by *N. cinerea* specimens in Pernambuco, Northeastern Brazil. *N. cinerea* nymphs were fed with lettuce samples for 48 h. A negative control group received fish feed hydrated in distilled water, while a positive control was exposed to cyclophosphamide 2 mg/mL within the feed. DNA damage was assessed through two independent experiments using the comet assay on hemolymph cells; results were classified on a scale from 0 to 4 and quantified using Damage Index and Damage Frequency. After exposure and comet assay performance, the hemocytes of *N. cinerea* demonstrated significant DNA damage within all samples consumed, including the positive control, suggesting the good sensibility of the model and the potential presence of genotoxic compounds on the crop samples. *N. cinerea* appeared as a sensitive bioindicator for the assessment of DNA damage, offering a novel and potentially valuable tool for assessing a variety of stressors on the DNA. Our findings indicate potential risks associated with lettuce consumption by non‐human organisms. Additionally, further investigations integrating water, soil and the vegetable itself are encouraged to verify potentially toxic elements in the crop.

## 1 Introduction

Fueled by a significant increase in agricultural output, Brazil's economy has experienced substantial growth in recent years, positioning the country as one of the world's leading food producers [1]. However, this rapid expansion has been accompanied by adverse environmental and public health consequences, as the negative impacts of agro‐industrial activities have become increasingly evident [1, 2]. This shift has resulted in the degradation of native biota, with their increasing contact with pesticides and has introduced new risks to human health [3, 4].

Among the risks, the extensive use of biocides has raised significant concerns due to their potential genotoxic effects. Human exposure to these compounds occurs through multiple environmental pathways, including air, water, soil, and food, as well as through contact with pesticide degradation products [5]. One of the most critical outcomes of such exposure is genotoxicity, which compromises the integrity of genetic material, potentially leading to long‐term health consequences [6].

In this context, the development and improvement of sensitive methodologies for biomonitoring genotoxic effects, particularly those affecting food safety, are essential [7]. To assess the biological effects of such contaminants without resorting to complex organisms like rodents or humans, the use of invertebrate bioindicators has gained prominence. The speckled cockroach ( *Nauphoeta cinerea* Olivier 1789), an arthropod belonging to the Blaberidae family, has proven to be an effective model in research of toxicological [8, 9], neurotoxicological [7, 10], pharmacological, and even edible compounds such as caffeine [10, 11].

Although, to the best of our knowledge, no study has been performed yet to analyze the genotoxic sensibility of this organism, even though DNA integrity analysis should come as a supplementary and important method for ecotoxicology research. Furthermore, the need for genetic evaluation is being neglected, mainly when the production of reactive oxygen species (ROS) is so intensively verified in *N. cinerea* [7, 8, 12]. In addition, this species is easily maintained under laboratory conditions, exhibits a rapid and comprehensive reproductive cycle, and has well‐characterized physiological and neurological features, making it a versatile experimental model for comparative studies with both invertebrates and vertebrates [7, 13].

Among the variety of consumable vegetables, lettuce ( *Lactuca sativa* ) is a globally cultivated crop and considered a relevant subject for genotoxic evaluation. Pollutants, including heavy metals and organic microcontaminants (OMCs), can accumulate in lettuce through chemical fertilization, fungicide application, and irrigation with contaminated water [14, 15, 16]. Additionally, studies conducted in Brazil, a country with high lettuce consumption, have reported the significant presence of potentially toxic elements (PTEs) on this vegetable [17].

In this context, the present study aimed to evaluate the potential of *N. cinerea* to be used as a bioindicator for genotoxicity through the implementation of the comet assay in this organism. The comet assay is chosen due to its high sensitivity and broad applicability in detecting DNA damage induced by pesticides and other environmental stressors [18, 19, 20, 21, 22, 23]. Additionally, we investigated the effects that the ingestion of *L. sativa* samples commercially available in Pernambuco, Northeastern Brazil, would have on the mentioned organism.

## 2 Materials and Methods

### 2.1 Specimen Collection and Rearing Conditions

*N. cinerea* is a cockroach species characterized by its mottled grayish coloration and distinctive wing patterns [24]. It is nocturnal, and commonly found in environments associated with human activity [25]. Specimens used in this study were obtained from the Laboratory of Toxinology Applied to Pharmacology and Scorpion Behavior at the Centro Acadêmico de Vitória, Universidade Federal de Pernambuco (UFPE), and maintained under controlled laboratory conditions. The insects were reared in plastic containers (30 × 20 cm) at a constant temperature of 24°C–25°C, with a 14:10 h light/dark photoperiod. They had continuous access to water and were fed weekly with commercial fish feed [26].

### 2.2 Experimental Design

The experiments were conducted in two independent phases: January 2023 and January 2024. Fresh *L. sativa* samples were collected from two public markets in Vitória de Santo Antão (VSA), one collected directly from a non‐organic cultivation site in Natuba (VSA) and one collected in an organic site in Pombos (POBO), totaling 4 samples, both located in the *Zona da Mata* region, Pernambuco, Brazil.

*N. cinerea* nymphs were divided into six experimental groups, each comprising 18 individuals distributed in three replicates (6 nymphs per replicate) [9]. The nymphs were housed in plastic containers (15 × 10 cm) with perforated lids to ensure proper ventilation. Prior to the feeding phase, all nymphs underwent a seven‐day fasting period to standardize their metabolic state. Subsequently, they were fed *ad libitum* for 48 h with in natura lettuce samples, according to the following treatments: NAT: Lettuce from the Natuba cultivation region (VSA); POBO: Organic lettuce from the Poço do Boi cultivation region (Pombos); SM1: Lettuce purchased from the 1st public market in VSA; SM2: Lettuce purchased from the 2nd public market in VSA.

Two control groups were established to validate the assay:

*Negative Control (CO−)*: Nymphs fed 2 g of crushed fish feed hydrated with 4 mL of distilled water.

*Positive Control (CO+)*: Nymphs subjected to the same fasting protocol as CO−, followed by feeding with fish feed hydrated with cyclophosphamide (a known genotoxic compound) diluted in distilled water at a concentration of 2 mg/mL [27].

With a total of 216 individuals analyzed throughout the experiments.

### 2.3 Comet Assay Procedure

The comet assay was conducted on *N. cinerea* nymphs following the protocol described by Silva et al. [23] for scorpions, with specific adaptations here described to suit the experimental model used in this study.

#### 2.3.1 Sample Preparation and Hemolymph Extraction

After the 48‐h feeding period [28] with *L. sativa* , the nymphs were retrieved from their containers and subjected to a brief cooling process (~2 min) to reduce metabolic activity and facilitate handling without interfering with the DNA integrity [29]. According to established protocols used in genotoxic research [21, 23, 30], and to be replicable to perform together with biochemical analysis [9], hemolymph was extracted from pools of six nymphs (constituting one replicate) even though it can be extracted individually, depending on the aim of the study [29]. Hemolymph was released through the amputation of locomotor appendages and bilateral incisions in the thorax. The hemolymph was collected in concave glass plates (Kline dishes) containing 200 μL of ethylenediaminetetraacetic acid (EDTA), avoiding agglutination of hemocytes and subsequent degradation of DNA (prepared with 3 g NaOH, 14.89 g EDTA, and 200 mL distilled water, adjusted to pH 10) [31].

The collected hemolymph was transferred to 1.5 mL microcentrifuge tubes containing an additional 300 μL of EDTA solution and centrifuged at 3000 rpm for 9 min. All subsequent procedures were performed under red light in a dark room to minimize potential DNA damage caused by light exposure [29].

#### 2.3.2 Slide Preparation

Post‐centrifugation, 60 μL of the supernatant from each replicate was mixed with 100 μL of 0.5% low‐melting‐point agarose (LM agarose). This mixture was immediately dispensed onto pre‐cleaned glass slides coated with a base layer of 1.5% standard agarose. The slides were covered with 24 × 60 mm coverslips and placed at 4°C to allow the agarose to solidify briefly. For each treatment group, a total of 12 slides were prepared (four slides per replicate, with three replicates per group).

#### 2.3.3 Cell Lysis and Electrophoresis

Following solidification, the coverslips were gently removed, and the slides were immersed in a lysis solution (2.5 M NaCl, 1 M EDTA, 100 mM NaOH, 1% Tris pH 10, 1% Triton X‐100, 10% dimethyl sulfoxide) at 4°C for 48 h.

After lysis, the slides were transferred to a horizontal electrophoresis tank (40 cm) filled with alkaline buffer solution (1 M NaOH, 200 mM EDTA, pH > 13) and incubated for 20 min on ice to allow DNA unwinding. Electrophoresis was then performed at 40 V (1 V/cm), 300 mA, and 120 W for 20 min on ice. Subsequently, neutralization and fixation steps were carried out as described by Silva et al. [23]. The prepared slides were stored at 4°C in light‐protected containers until microscopic analysis.

#### 2.3.4 DNA Damage Assessment

For the evaluation of DNA damage, nucleoids were stained with 50 μL of GelRed dye (Biotium), diluted 1:500 in distilled water. The slides were examined under a Zeiss Axio Imager M2 fluorescence microscope at 400× magnification using a 546 nm filter [30].

**FIGURE 1:**
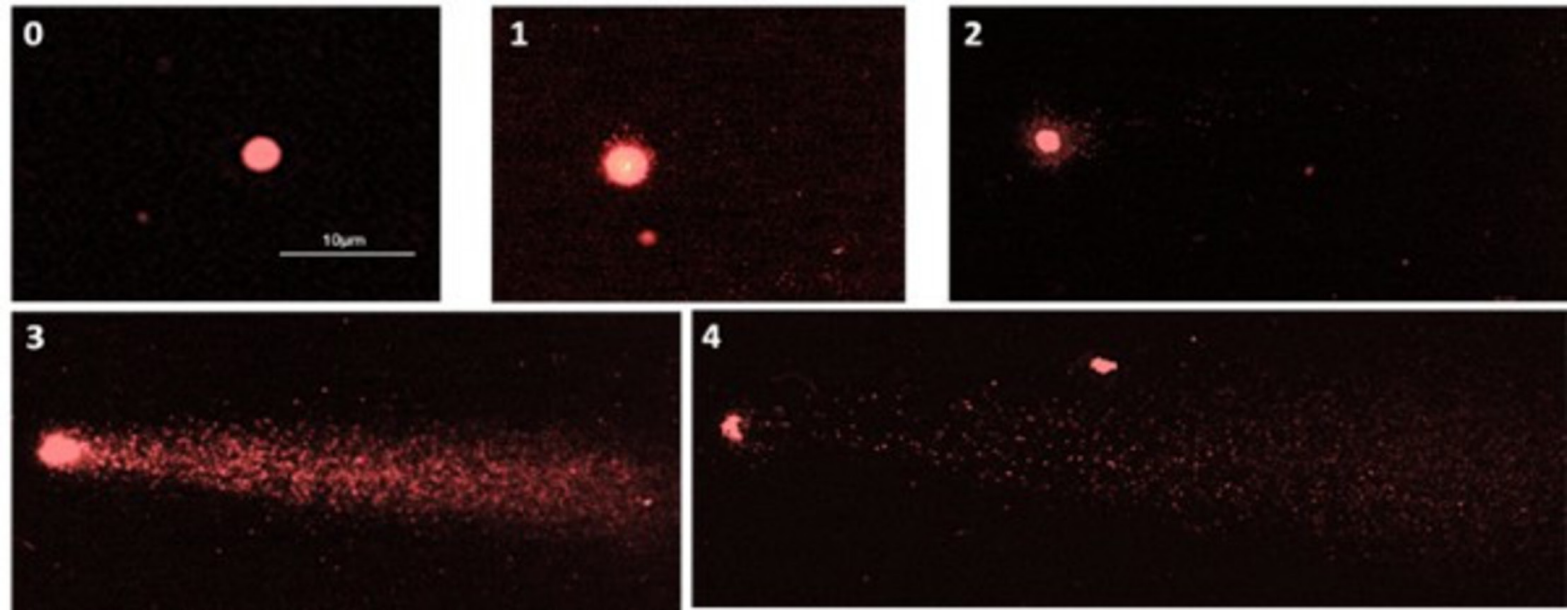
Visual scoring of DNA damage (0–4) based on comet appearance in hemocyte of *Nauphoeta cinerea* following comet assay, stained with GelRed.

DNA damage was assessed by a single person, with a blind analysis of the slides [6, 23]. Nucleoids were classified (visual scoring) on a scale from 0 to 4, where 0 indicated no detectable damage and 4 represented severe damage (long comet tails with low head integrity) (Figure 1). For each replicate, 100 nucleoids were analyzed (50 per slide, across two slides), totaling 300 nucleoids per treatment group.

#### 2.3.5 Calculation of Damage Index (DI) and Damage Frequency (DF%)

The values obtained independently from the experiments carried out in January 2023 and January 2024 were combined, and the means were submitted to statistical analysis (Table S1). The extent of DNA damage was quantified using two parameters: the Damage Index (DI) and Damage Frequency (DF%), as follows:

$$\mathrm{DI}=0\times \left(N0\;\right)+1\times \left(N1\;\right)+2\times \left(N2\;\right)+3\times \left(N3\;\right)+4\times \left(N4\;\right)$$

$$\mathrm{DF}\left(\%\right)=\left(\frac{\mathrm{NT}-N0}{\mathrm{NT}}\right)\times 100$$

Full description of the formulas is presented in [32]. The DI and DF% values obtained for each treatment group were subjected to analysis of variance (ANOVA) to detect significant differences. When ANOVA results were significant, pairwise comparisons were performed using the Bonferroni post hoc test. All statistical analyses were conducted using STATA software version 14.2, with a significance level of *p* < 0.05.

## 3 Results and Discussion

**FIGURE 2:**
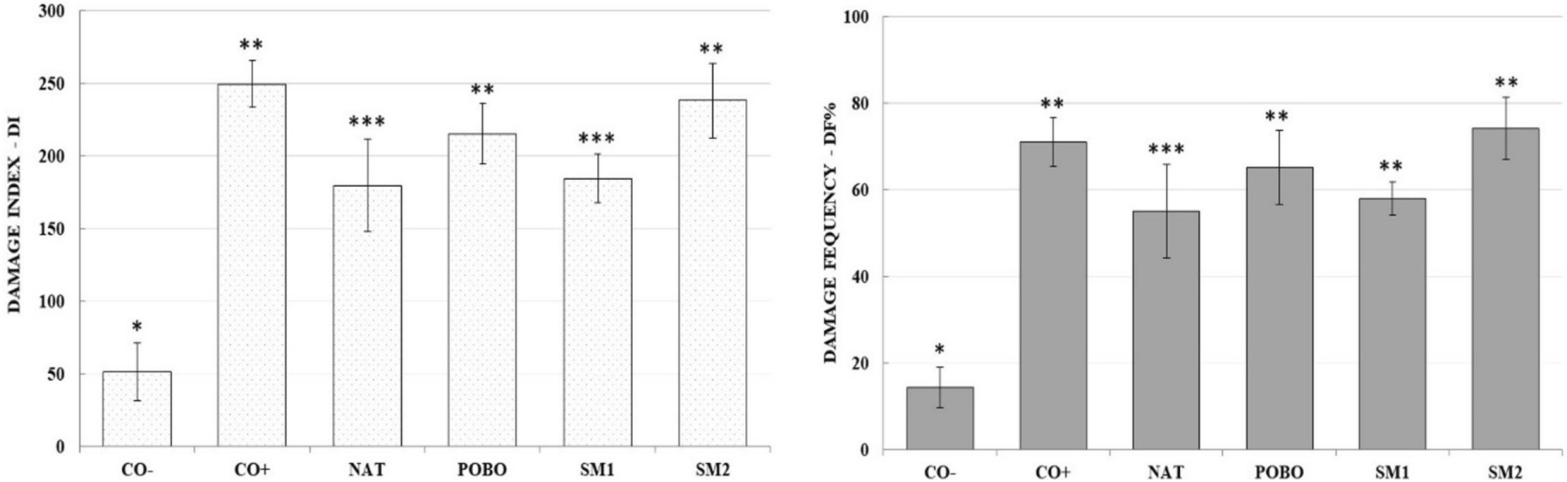
Mean damage index (DI) and mean damage frequency (DF%) calculated for each treatment group of *Nauphoeta cinerea* fed with lettuce samples, including negative and positive controls.

The results of the comet assay performed on *N. cinerea* nymphs fed with different lettuce samples, as well as the negative control (CO−) and positive control (CO+) groups, are presented in Figure 2. Pairwise comparisons of the mean Damage Index (DI) and Damage Frequency (DF%) across treatments are detailed in Table 1. Additionally, comprehensive experimental data for each replicate, collected during the two independent experimental periods (2023 and 2024), are provided as Table S2.

**TABLE 1:** Pairwise post‐test of Bonferroni comparisons of the combined experiments means Damage Index (DI) and Damage Frequency (DF%) in *Nauphoeta cinerea* . Values below the diagonal represent DI, while values above the diagonal represent DF%.

	Groups	CO−	CO+	NAT	POBO	SM1	SM2	Statistical analysis—DF%
	CO−		0.0001*	0.001*	0.0001*	0.0001*	0.0001*	|*F* = 59.6| |df = 17| |Prob > chi2 = 0.813|
	CO+	0.0001*		0.026*	1.000	0.104*	1.000	
	NAT	0.0001*	0.002*		0.386	1.000	0.007*	
	POBO	0.0001*	0.313	0.258		1.000	0.658	
	SM1	0.0001*	0.004*	1.000	0.514		0.024*	
	SM2	0.0001*	1.000	0.010*	1.000	0.020*		
Statistical analysis—DI	|*F* = 61.81|df = 17|Prob > chi2 = 0.941|							

*Note:* CO− (negative control); CO+ (positive control); NAT (Lettuce from Natuba cultivation region in Vitória de Santo Antão); POBO (Lettuce from Poço do Boi cultivation region in Pombos); SM1 (Lettuces from the public street market in Vitória de Santo Antão—1st selling point); SM2 (Lettuces from the public street market in Vitória de Santo Antão—2nd selling point).

* Statistically significant differences (*p* ≤ 0.05).

As hypothesized, individuals in the CO− group, which were fed ground fish feed, exhibited a relatively low frequency of damaged nucleoids. Conversely, the CO+ group, exposed to fish feed hydrated with a cyclophosphamide solution (2 mg/mL), displayed significantly elevated DI and DF% values. Both CO− and CO+ differed statistically in the Damage Index and the Damage Frequency (*p* < 0.0001).

The data obtained for the controls are similar to those obtained in other invertebrate organisms submitted to a similar protocol, like the well established genotoxic model *Drosophila melanogaster* [21, 32, 33] or the scorpion *Tityus pusillus* [23]. This methodology stands on its feet due to its potential to show a momentary status of the nucleus of a cell undergoing stress; further genotoxic methodologies, such as the micronucleus assay, should be incorporated and implemented to verify the establishment or repair of these breaks, which are the initial event for carcinogenesis [6].

Before we discuss more profoundly our findings, we acknowledge that the lack of chemical analysis (metal or pesticide levels) within lettuce samples here tested leaves an important gap in the direct cause of the genotoxicity seen in *N. cinerea* and must be considered in further applied studies. Although we couldn't establish a partnership to analyze the samples. So, reinforcing that our focus, as exploratory research, was to validate the comet assay in the organism for future use in the ecotoxicology field.

All lettuce samples, regardless of their source, induced genotoxic effects in *N. cinerea* . Among the tested samples, the NAT treatment exhibited the lowest mean DI and DF% values. However, the POBO group, derived from a cultivation site mentioned as organic by the producer, showed no significant difference in DI or DF% compared to conventionally sourced lettuce samples (NAT). Studies have pointed out that areas of organic cultivation may present chemical compounds as a hereditary factor from past crops [34].

Lettuce samples from urban street markets (SM1 and SM2), both commercialized in Vitória de Santo Antão, presented also high DI and DF% values. These samples exhibited significant differences in genotoxicity, reflecting inherent variability in agricultural practices at their respective cultivation or handling processes. The lack of detailed information regarding pesticide use, water source and cultivation conditions for these samples further underscores the potential influence of unregulated agricultural practices on the genotoxic outcomes found here.

It is suggested that variations in cultivation conditions do influence lettuce crops, which are reflected on the observed genotoxicity. Several environmental factors, beyond the direct application of pesticides, can contribute to the contamination of food crops. For instance, Araújo Júnior et al. [35] provided evidence that the use of poor‐quality irrigation water can lead to soil degradation and adversely affect the quality of irrigated crops. Additionally, Gupta et al. [36] highlighted that the chemical composition of the soil plays a critical role in plant health, as vegetables absorb both micro and macronutrients from the surrounding soil environment, along with potential contaminants.

Pesticide residues clearly cannot be discarded in explaining the genotoxicity observed. The area of NAT is known for crop cultivation with the indiscriminate use of agrochemicals and pesticides [37]. In a study by Ripke et al. [38], pesticide residues such as azoxystrobin, deltamethrin, imidacloprid, and glyphosate, commonly used in Brazilian agricultural practices, were surveyed in both conventional and organically cultivated lettuce. Although pesticides residues were detected only in conventionally grown samples and at legal limits established by Brazilian regulations, organically cultivated crops have presented contamination levels as well in other regions around the globe [34, 38, 39]. With that said, the response of living organisms to such contamination has been underestimated [40]. This underscores the need for integrated analysis with both chemical and biological assays, so ascertaining food and environmental safety not only in developing countries like Brazil, but in every place submitted to crop cultivation.

Alternatively, studies performed in 2013 have pointed out the presence, above safety limits, of azoxystrobin and other agrochemicals in the groundwater and soil of NAT cultivation area (Nascimento, *apud* [37]), which would justify the genotoxicity of the lettuce there obtained.

Nevertheless, even low concentrations of such compounds can exert cumulative genotoxic effects, particularly when combined with other environmental stressors. In fact, according to the State Environmental Agency of Pernambuco [41], the reference concentrations of potentially toxic elements (PTEs) in soils are expected to be substantially lower than those determined in a more recent parallel analyses performed by our research group on the NAT soils. Specifically, CPRH establishes reference limits of 13 mg/kg for Pb, 35 mg/kg for Zn, 9 mg/kg for Ni, and 5 mg/kg for Cu. In contrast, the concentrations obtained by our analysis were: Pb = 23.3 mg/kg; Zn = 235.1 mg/kg; Ni = 16.2 mg/kg; Cu = 79.9 mg/kg (see details of the analysis in Table S3). Considerably exceeding these thresholds, indicating a potential contamination concern in soils currently under cultivation. For instance, chronic exposure to Cu and Zn induces neurolocomotor depletion and increases concentration of oxidative stress biomarkers [42]. Complementary, the intake of enriched Pb clover can induce high DNA damage values, reinforcing the potential of contaminated food ingestion in causing genotoxicity [43].

Therefore, it is conceivable that the elevated genotoxicity levels observed in *N. cinerea* fed with in natura lettuce samples result from a combination of multiple contamination sources, including poor water quality, high concentrations of soil pollutants, and pesticide residues [37]. This is reinforced when the combination of lettuce and vegetables has been used to feed the cockroach *Gromphadorinha coquereliana*, and it didn't induce high genotoxicity as it was seen in this research [29]. The multifactorial nature of contamination highlights the importance of adopting an integrated and comprehensive approach when evaluating food safety and environmental health.

Our study underscores the need to assess environmental factors beyond the food product itself, as crops can be contaminated through indirect pathways such as irrigation, soil quality, and post‐harvest handling [44]. Monitoring the quality of water used in organic farming is particularly crucial, as even pesticide‐free crops can accumulate toxic substances from environmental sources [34, 45]. Although the organic lettuce producer claimed that the samples were free of pesticides, soil treatments and remediation strategies may inadvertently lead to the accumulation of PTEs, which can subsequently contaminate crops and chronically harm the native biota [1, 3, 14]. Besides that, soil health is a key factor in guaranteeing food security and a truly organic management strategy [46].

## 4 Conclusions

Based on the innovative findings of this study, it is suggested that the model organism *N. cinerea* is sensitive to the comet assay methodology and the ingestion of *L. sativa* , exhibiting varying levels of genetic damage. These results highlight the potential of *N. cinerea* as a bioindicator for assessing genotoxicity, and it is hypothesized that the lettuce cultivation sites are under contamination, which is reflected on the tested vegetables and was signaled by the *N. cinerea* organisms. However, to establish a definitive link between the observed genotoxic effects and specific environmental contaminants, further investigations are warranted. Future studies should focus on the chemical analysis of lettuce samples, as well as the water and soil used in their cultivation. Identifying the potential causative agents of genetic damage will be essential for understanding the complex interactions between agricultural practices, environmental conditions, and food safety. This approach will contribute to the development of more effective strategies for monitoring and mitigating risks associated with food contamination.

## Author Contributions

**Aleson Aparecido da Silva:** conceptualization, methodology, validation, formal analysis, investigation, writing – original draft, writing – review and editing, visualization. **Eduardo Henrique da Silva Melo:** writing – original draft, methodology, visualization. **Maria Gislaine Pereira:** methodology, writing – review and editing. **Érima Maria de Amorim:** methodology. **Claudia Rohde:** conceptualization, resources, data curation, writing – review and editing, supervision, project administration, funding acquisition.

## Funding

This research was partially funded by the Fundação de Amparo à Ciência e Tecnologia do Estado de Pernambuco (FACEPE) under the process number IBPG‐1784‐2.02/21; and by the Coordenação de Aperfeiçoamento de Pessoal de Nível Superior—Brasil (CAPES)—Finance Code 001.

## Conflicts of Interest

The authors declare no conflicts of interest.

## Supporting information


**Table S1:** Means of Damage Index, and Damage Frequency assessed in three replicates collected during two independent experiments (1‐January 2023; 2‐January 2024). Results from both experiments were combined, and replicates are labeled as “a”, “b”, and “c.”
**Table S2:** Values of Mean and Standard Deviation (SD) of damage levels (0 to 4), damage index and damage frequency assessed in the three replicates of Nauphoeta cinerea nymphs (each with six individuals) feed with lettuce samples collected in two moments (1 = January 2023; 2 = January 2024). Replicates are labeled as a, b, and c.
**Table S3:** Values of the chemical analysis of five soil samples obtained from Natuba cultivation area (NAT) through the Energy dispersive X‐ray fluorescence (EDXRF), plus the central tendency values. Final values of central tendency are expressed in mg/kg.

## Data Availability

The data that supports the findings of this study are available in the Supporting Information of this article.

## References

[1] C. L. F. Fernandes , L. M. Volcão , P. F. Ramires , R. R. D. Moura , and F. M. R. D. S. Júnior , “Distribution of Pesticides in Agricultural and Urban Soils of Brazil: A Critical Review,” Environmental Science: Processes and Impacts 22 (2020): 256–270, 10.1039/C9EM00433E. 31984396

[2] H. K. Bayabil , F. T. Teshome , and Y. C. Li , “Emerging Contaminants in Soil and Water,” Frontiers in Environmental Science 10 (2022): 873499, 10.3389/fenvs.2022.873499.

[3] W. Ahmad , R. D. Alharthy , M. Zubair , M. Ahmed , A. Hameed , and S. Rafique , “Toxic and Heavy Metals Contamination Assessment in Soil and Water to Evaluate Human Health Risk,” Scientific Reports 11 (2021): 17006, 10.1038/s41598-021-94616-4. 34417479 PMC8379239

[4] L. Rui , L. ShengCai , L. Jia , and Y. HuaiQing , “Using Micronuclei Test and Single Cell Gel Electrophoresis Assay to Evaluate Genotoxicity of Pesticides to *Pardosa Astrigera* L. Koch,” Zhongguo Shengtai Nongye Xuebao = Chinese Journal of Eco‐Agriculture 20 (2012): 58–62, 10.3724/SP.J.1011.2012.00058.

[5] C. Bolognesi , “Genotoxicity of Pesticides: A Review of Human Biomonitoring Studies,” Mutation Research 543 (2003): 251–272, 10.1016/s1383-5742(03)00015-2. 12787816

[6] P. Møller , “The Alkaline Comet Assay: Towards Validation in Biomonitoring of DNA Damaging Exposures,” Basic and Clinical Pharmacology and Toxicology 98, no. 4 (2006): 336–345, 10.1111/j.1742-7843.2006.pto_167.x. 16623855

[7] I. A. Adedara , K. A. Mohammed , J. Canzian , et al., “ *Nauphoeta cinerea* as an Emerging Model in Neurotoxicology,” Advances in Neurotoxicology 9 (2023): 181–196, 10.1016/bs.ant.2023.01.004. 37389201 PMC10310038

[8] I. A. Adedara , K. A. Mohammed , O. F. Da‐Silva , et al., “Utility of Cockroach as a Model Organism in the Assessment of Toxicological Impacts of Environmental Pollutants,” Environmental Advances 8 (2022): 100195, 10.1016/j.envadv.2022.100195. 35992224 PMC9390120

[9] P. S. Pereira , A. R. Costa , T. J. S. Oliveira , et al., “Neurolocomotor Behavior and Oxidative Stress Markers of Thiazole and Thiazolidinedione Derivatives Against *Nauphoeta Cinerea* ,” Antioxidants (Basel) 11 (2022): 420, 10.3390/antiox11020420. 35204302 PMC8869355

[10] I. A. Adedara , I. O. Awogbindin , B. A. Afolabi , B. O. Ajayi , J. B. T. Rocha , and E. O. Farombi , “Hazardous Impact of Diclofenac Exposure on the Behavior and Antioxidant Defense System in *Nauphoeta cinerea* ,” Environmental Pollution 265 (2020): 115053, 10.1016/j.envpol.2020.115053. 32806419

[11] C. S. da Silva , R. d. C. G. Lima , O. O. Elekofehinti , et al., “Caffeine‐Supplemented Diet Modulates Oxidative Stress Markers and Improves Locomotor Behavior in the Lobster Cockroach *Nauphoeta cinerea* ,” Chemico‐Biological Interactions 282 (2018): 77–84, 10.1016/j.cbi.2018.01.011. 29339219

[12] I. A. Adedara , O. O. Abioye , G. T. Oyedele , et al., “Perfluorooctanoic Acid Induces Behavioral Impairment and Oxidative Injury in *Nauphoeta cinerea* Nymphs,” Environmental Science and Pollution Research 30 (2023): 110340–110351, 10.1007/s11356-023-30156-w. 37783994

[13] I. A. Adedara , D. B. Rosemberg , D. de Souza , et al., “Neurobehavioral and Biochemical Changes in *Nauphoeta cinerea* Following Dietary Exposure to Chlorpyrifos,” Pesticide Biochemistry and Physiology 130 (2016): 22–30, 10.1016/j.pestbp.2015.12.004. 27155480

[14] M. Malandrino , O. Abollino , S. Buoso , A. Giacomino , C. La Gioia , and E. Mentasti , “Accumulation of Heavy Metals From Contaminated Soil to Plants and Evaluation of Soil Remediation by Vermiculite,” Chemosphere 82 (2011): 169–178, 10.1016/j.chemosphere.2010.10.028. 21055788

[15] A. Margenat , V. Matamoros , S. Díez , N. Cañameras , J. Comas , and J. M. Bayona , “Occurrence and Bioaccumulation of Chemical Contaminants in Lettuce Grown in Peri‐Urban Horticulture,” Science of the Total Environment 637–638 (2018): 1166–1174, 10.1016/j.scitotenv.2018.05.035. 29801210

[16] V. Matamoros , A. M. Rendón‐Mera , B. Piña , et al., “Metabolomic and Phenotypic Implications of the Application of Fertilization Products Containing Microcontaminants in Lettuce ( *Lactuca sativa* ),” Scientific Reports 11 (2021): 9701, 10.1038/s41598-021-89058-x. 33958645 PMC8102503

[17] C. N. Lange , B. M. Freire , L. R. Monteiro , M. E. B. Cotrim , and B. L. Batista , “Potentially Toxic Elements in Urban‐Grown Lettuce: Effectiveness of Washing Procedures, Risk Assessment, and Isotopic Fingerprint,” Plants 13, no. 19 (2024): 2807, 10.3390/plants13192807. 39409676 PMC11479218

[18] M. Bajpayee , A. Kumar , and A. Dhawan , “The Comet Assay: Assessment of In Vitro and In Vivo DNA Damage,” in Genotoxicity Assessment: Methods and Protocols, Methods in Molecular Biology, ed. A. Dhawan and M. Bajpayee (Springer, 2019), 237–257, 10.1007/978-1-4939-9646-9_12. 31473963

[19] A. R. Collins , V. L. Dobson , M. Dušinská , G. Kennedy , and R. Štětina , “The Comet Assay: What Can It Really Tell Us?,” Mutation Research, Fundamental and Molecular Mechanisms of Mutagenesis 375 (1997): 183–193, 10.1016/S0027-5107(97)00013-4. 9202728

[20] J. Lapuente , J. Lourenço , S. A. Mendo , et al., “The Comet Assay and Its Applications in the Field of Ecotoxicology: A Mature Tool That Continues to Expand Its Perspectives,” Frontiers in Genetics 6 (2015): 180, 10.3389/fgene.2015.00180. 26089833 PMC4454841

[21] M. G. Pereira , E. M. D. Amorim , A. A. Silva , D. Guimarães‐Silva , A. M. Esteves , and C. Rohde , “Evidences of Radioresistance in *Drosophila melanogaster* From Northeastern Brazil,” International Journal of Radiation Biology 101, no. 2 (2025): 164–173, 10.1080/09553002.2024.2440858. 39689110

[22] S. L. Santana , C. J. Verçosa , Í. F. Araújo Castro , et al., “ *Drosophila melanogaster* as Model Organism for Monitoring and Analyzing Genotoxicity Associated With City Air Pollution,” Environmental Science and Pollution Research 25 (2018): 32409–32417, 10.1007/s11356-018-3186-5. 30229497

[23] A. A. Silva , É. M. Amorim , M. G. Pereira , et al., “Genotoxic Effects of Anthropogenic Environments in the Leaf Litter‐Dwelling Scorpion *Tityus pusillus* Pocock, 1893 (Scorpiones; Buthidae),” Mutation Research, Genetic Toxicology and Environmental Mutagenesis 887 (2023): 503585, 10.1016/j.mrgentox.2023.503585. 37003646

[24] S. Bouchebti , V. Durier , C. Pasquaretta , C. Rivault , and M. Lihoreau , “Subsocial Cockroaches *Nauphoeta cinerea* Mate Indiscriminately With Kin Despite High Costs of Inbreeding,” PLoS One 11 (2016): e0162548, 10.1371/journal.pone.0162548. 27655156 PMC5031396

[25] M.‐H. Lizée , B. Barascud , J.‐P. Cornec , and L. Sreng , “Courtship and Mating Behavior of the Cockroach *Oxyhaloa Deusta* [Thunberg, 1784] (Blaberidae, Oxyhaloinae): Attraction Bioassays and Morphology of the Pheromone Sources,” Journal of Insect Behavior 30 (2017): 674–694, 10.1007/s10905-017-9648-7.

[26] B. Silva and A. Pelli , “Metodologia para criação de três espécies de Blattaria Burmeister, 1829: Nauphoeta Cinerea (Olivier, 1789), Blaberus Giganteus (Linnaeus, 1758) e Gromphadorhina Portentosa (Schaum, 1853),” Acta Biologica Brasiliensia 3 (2020): 14–21, https://crbio04.gov.br/acta/acta‐biologica‐brasiliensia‐vol‐3‐no1/.

[27] L. P. Wachsmuth , M. T. Patterson , M. A. Eckhaus , D. J. Venzon , and C. G. Kanakry , “Optimized Timing of Post‐Transplantation Cyclophosphamide in MHC‐Haploidentical Murine Hematopoietic Cell Transplantation,” Biology of Blood and Marrow Transplantation 26 (2020): 230–241, 10.1016/j.bbmt.2019.09.030. 31586477 PMC7590501

[28] M. Baniardalani , A. A. Rahimian , A. Saghafipour , H. R. Basseri , M. Kababian , and J. Nejati , “Toxicity of Imidacloprid and Chlorpyrifos Against German Cockroaches *Blattella germanica* ,” International Journal of One Health 5 (2019): 107–112, 10.14202/IJOH.2019.107-112.

[29] J. Lubawy , V. Daburon , S. Chowański , M. Słocińska , and H. Colinet , “Thermal Stress Causes DNA Damage and Mortality in a Tropical Insect,” Journal of Experimental Biology 222, no. 23 (2019): JEB213744, 10.1242/jeb.213744. 31672731

[30] É. M. Amorim , S. L. de Santana , A. S. Silva , et al., “Genotoxic Assessment of the Dry Decoction of *Myracrodruon Urundeuva* Allemão (Anacardiaceae) Leaves in Somatic Cells of *Drosophila Melanogaster* by the Comet and SMART Assays,” Environmental and Molecular Mutagenesis 61, no. 3 (2020): 329–337, 10.1002/em.22332. 31489703

[31] G. Banfi , G. L. Salvagno , and G. Lippi , “The Role of Ethylenediamine Tetraacetic Acid (EDTA) as *in Vitro* Anticoagulant for Diagnostic Purposes,” Clinical Chemistry and Laboratory Medicine 45, no. 5 (2007): 565–576, 10.1515/CCLM.2007.110. 17484616

[32] C. Nascimento‐Silva , E. F. Carmo‐Neto , S. L. Santana , et al., “Accessing the Health Risk of Ingestion of Surface Water From Lucrécia and Parelhas Dams in Northeast Brazil Using the Sentinel Organism *Drosophila melanogaster* ,” Bulletin of Environmental Contamination and Toxicology 112, no. 1 (2023): 12, 10.1007/s00128-023-03838-x. 38093100

[33] C. J. Verçosa , A. V. Moraes Filho , Í. F. Araújo Castro , et al., “Validation of Comet Assay in Oregon‐R and Wild Type Strains of *Drosophila melanogaster* Exposed to a Natural Radioactive Environment in Brazilian Semiarid Region,” Ecotoxicology and Environmental Safety 141 (2017): 148–153, 10.1016/j.ecoenv.2017.03.024. 28340370

[34] J. Riedo , F. E. Wettstein , A. Rösch , et al., “Widespread Occurrence of Pesticides in Organically Managed Agricultural Soils—The Ghost of a Conventional Agricultural Past?,” Environmental Science and Technology 55, no. 5 (2021): 2919–2928, 10.1021/acs.est.0c06405. 33534554

[35] G. N. Araújo Júnior , J. E. F. de Morais , L. S. B. Souza , A. J. Steidle Neto , G. G. L. Araujo , and T. G. F. Silva , “Use of Lower Quality Water in Irrigated Agriculture and Effects on Forages With Productive Potential in Semiarid Regions: A Review,” Environmental Processes 10 (2023): 44, 10.1007/s40710-023-00655-6.

[36] N. Gupta , K. K. Yadav , V. Kumar , S. Kumar , R. P. Chadd , and A. Kumar , “Trace Elements in Soil‐Vegetables Interface: Translocation, Bioaccumulation, Toxicity and Amelioration—A Review,” Science of the Total Environment 651 (2019): 2927–2942, 10.1016/j.scitotenv.2018.10.047. 30463144

[37] J. G. d. C. Marques , M. R. C. C. Lyra , R. M. C. M. d. O. Carvalho , R. M. do Nascimento , J. A. A. da Silva , and S. M. G. L. Montenegro , “Comparação Entre Índices de Potencial de Lixiviação Para Agrotóxicos Utilizados na Sub‐Bacia do Natuba, Vitória de Santo Antão‐Pernambuco,” Águas Subterrâneas 33 (2019): 58–67, 10.14295/ras.v33i1.29239.

[38] M. O. Ripke , J. A. Lutinski , and V. S. Corralo , “Segurança de Alimentos Comercializados em Feiras Livres: Análise de Resíduos de Agrotóxicos Em Alface (*Lactuca sativa* L.),” Revista Brasileira de Ciências Ambientais 57 (2022): 467–476, 10.5327/Z2176-94781376.

[39] ANVISA, A.N. de V.S , “Programa de Análise de Resíduos de Agrotóxicos ‐ Relatório 2017 e 2018 — Agência Nacional de Vigilância Sanitária—Anvisa (Tecnical). Ministério da Saúde,” (2022), https://www.gov.br/anvisa/pt‐br/centraisdeconteudo/publicacoes/agrotoxicos/publicacoes/programa‐de‐analise‐de‐residuos‐de‐agrotoxicos‐relatorio‐2017‐e‐2018.pdf/view.

[40] B. B. Lauper , E. Anthamatten , J. Raths , M. Arlos , and J. Hollender , “Systematic Underestimation of Pesticide Burden for Invertebrates Under Field Conditions: Comparing the Influence of Dietary Uptake and Aquatic Exposure Dynamics,” ACS Environmental Au 2, no. 2 (2022): 166–175, 10.1021/acsenvironau.1c00023. 37101586 PMC10114668

[41] CPRH, Agência Estadual do Meio Ambiente , “Instrução Normativa n 7, de 07 de Julho de 2014. *Diário Oficial do Estado de Pernambuco*, Recife,” (2014), https://www2.cprh.pe.gov.br/wp‐content/uploads/2022/09/Instrucao‐Normativa‐CPRH‐No‐7‐2014.pdf.

[42] G. T. Oyedele , O. D. Atarase , A. A. Olaseni , J. B. T. Rocha , I. A. Adedara , and E. O. Farombi , “Impact of Chronic Exposure to Ternary Metal Mixtures on Behavioral and Cellular Responses in *Nauphoeta cinerea* Nymphs,” Environmental Entomology 54, no. 3 (2025): 409–420, 10.1093/ee/nvaf023. 40257999

[43] H. A. Yousef , A. Afify , H. M. Hasan , and A. A. Meguid , “DNA Damage in Hemocytes of *Schistocerca gregaria* (Orthoptera: Acrididae) Exposed to Contaminated Food With Cadmium and Lead,” Nature Science 2, no. 4 (2010): 292–297, 10.4236/ns.2010.24037.

[44] R. K. S. Araújo , A. R. D. Silva , N. R. Souza , and K. S. D. Melo , “Parasitological Analysis of Vegetables Sold in Supermarkets and Free Markets in the City of Taguatinga, Federal District, Brazil,” Revista de Patologia Tropical 51, no. 3 (2022): 225–234, 10.1515/CCLM.2007.110.

[45] M. Haghighi , P. Fang , and M. Pessarakli , “Effects of Ammonium Nitrate and Monosodium Glutamate in Waste Water on the Growth, Antioxidant Activity, and Nitrogen Assimilation of Lettuce (*Lactuca sativa* L.),” Journal of Plant Nutrition 38 (2015): 2217–2229, 10.1080/01904167.2015.1009102.

[46] J. W. Doran , S. I. Stamatiadis , and J. Haberern , “Soil Health as an Indicator of Sustainable Management,” Agriculture, Ecosystems and Environment 88 (2002): 107–110, 10.1016/S0167-8809(01)00250-X.

