# Supplementary material for: First Assessment of Genetic Damage in the Speckled Cockroach (Nauphoeta cinerea) After Consumption of Lettuce (Lactuca sativa) Cultivated and Commercialized in Northeastern, Brazil
Source: Environ Toxicol. 2026 Jan 5;41(9):682–9. doi: 10.1002/tox.70020 (PMC13436181; doi:10.1002/tox.70020)
Supplement: Supplementary file 1 [file TOX-41-682-s001.docx]

**SUPPLEMENTARY MATERIAL**

| Treatment groups combined | Damage Index (mean) | Damage Frequency % (mean) |
| --- | --- | --- |
| CO-.1a + CO-.2a | 62 | 17 |
| CO-.1b + CO-.2b | 64 | 17 |
| CO-.1c + CO-.2c | 29 | 9 |
| CO+.1a + CO+.2a | 237 | 65 |
| CO+.1b + CO+.2b | 249 | 73 |
| CO+.1c + CO+.2c | 264 | 75 |
| NAT.1a + NAT.2a | 182 | 54 |
| NAT.1b + NAT.2b | 170 | 53 |
| NAT.1c + NAT.2c | 187 | 59 |
| POBO.1a + POBO.2a | 194 | 57 |
| POBO.1b + POBO.2b | 230 | 69 |
| POBO.1c + POBO.2c | 222 | 71 |
| SM1.1a + SM1.2a | 173 | 55 |
| SM1.1b + SM1.2b | 204 | 61 |
| SM1.1c + SM1.2c | 177 | 59 |
| SM2.1a + SM2.2a | 221 | 69 |
| SM2.1a + SM2.2a | 244 | 77 |
| SM2.1a + SM2.2a | 250 | 77 |

Table S1. Means of Damage Index, and Damage Frequency assessed in three replicates collected during two independent experiments (.1-January 2023; .2-January 2024). Results from both experiments were combined, and replicates are labeled as ‘a’, ‘b’, and ‘c’.

CO- (Negative Control). CO+ (Positive Control). NAT (Lettuces from Natuba); POBO (Lettuces from Pombos); SM1 (Lettuces from the Street Market - 1º selling point); SM2 (Lettuces from the Street Market - 2º selling point).

Table S2. Values of Mean and Standard Deviation (SD) of damage levels (0 to 4), damage index and damage frequency assessed in the three replicates of *Nauphoeta cinerea* nymphs (each with six individuals) feed with lettuce samples collected in two moments (.1= January 2023; .2= January 2024). Replicates are labeled as a, b, and c.

| Treatment  groups | Damage levels | | | | | Damage  Index | | Damage  Frequency % | |
| --- | --- | --- | --- | --- | --- | --- | --- | --- | --- |
|  | 0 | 1 | 2 | 3 | 4 |  |  |  |  |
| CO-.1a | 87 | 0 | 1 | 2 | 10 |  | 48 | | 13 |
| CO-.1b | 85 | 0 | 0 | 0 | 15 |  | 60 | | 15 |
| CO-.1c | 96 | 0 | 1 | 3 | 0 |  | 11 | | 4 |
| Mean | 89.33 | 0.00 | 0.67 | 1.67 | 8.33 |  | 39.67 | | 10.67 |
| SD | 5.86 | 0.00 | 0.58 | 1.53 | 7.64 |  | 25.54 | | 5.86 |
| CO-.2a | 79 | 0 | 1 | 7 | 13 |  | 75 | | 21 |
| CO-.2b | 81 | 0 | 0 | 9 | 10 |  | 67 | | 19 |
| CO-.2c | 86 | 1 | 0 | 6 | 7 |  | 47 | | 14 |
| Mean | 82.00 | 0.33 | 0.33 | 7.33 | 10.00 |  | 63.00 | | 18.00 |
| SD | 3.61 | 0.58 | 0.58 | 1.53 | 3.00 |  | 14.42 | | 3.61 |
| CO+.1a | 31 | 0 | 6 | 20 | 43 |  | 244 | | 69 |
| CO+.1b | 15 | 1 | 15 | 32 | 37 |  | 275 | | 85 |
| CO+.1c | 14 | 0 | 3 | 37 | 46 |  | 301 | | 86 |
| Mean | 20.00 | 0.33 | 8.00 | 29.67 | 42.00 |  | 273.33 | | 80.00 |
| SD | 9.54 | 0.58 | 6.24 | 8.74 | 4.58 |  | 28.54 | | 9.54 |
| CO+.2a | 39 | 0 | 0 | 15 | 46 |  | 229 | | 61 |
| CO+.2b | 39 | 0 | 1 | 20 | 40 |  | 222 | | 61 |
| CO+.2c | 36 | 0 | 0 | 30 | 34 |  | 226 | | 64 |
| Mean | 38.00 | 0.00 | 0.33 | 21.67 | 40.00 |  | 225.67 | | 62.00 |
| SD | 1.73 | 0.00 | 0.58 | 7.64 | 6.00 |  | 3.51 | | 1.73 |
| NAT.1a | 51 | 0 | 1 | 33 | 15 |  | 161 | | 49 |
| NAT.1b | 39 | 5 | 1 | 50 | 5 |  | 177 | | 61 |
| NAT.1c | 25 | 1 | 0 | 70 | 4 |  | 227 | | 75 |
| Mean | 38.33 | 2.00 | 0.67 | 51.00 | 8.00 |  | 188.33 | | 61.67 |
| SD | 13.01 | 2.65 | 0.58 | 18.52 | 6.08 |  | 34.43 | | 13.01 |
| NAT.2a | 42 | 0 | 4 | 21 | 33 |  | 203 | | 58 |
| NAT.2b | 55 | 0 | 1 | 15 | 29 |  | 163 | | 45 |
| NAT.2c | 58 | 0 | 2 | 18 | 22 |  | 146 | | 42 |
| Mean | 51.67 | 0.00 | 2.33 | 18.00 | 28.00 |  | 170.67 | | 48.33 |
| SD | 8.50 | 0.00 | 1.53 | 3.00 | 5.57 |  | 29.26 | | 8.50 |
| POBO.1a | 37 | 0 | 0 | 46 | 17 |  | 206 | | 63 |
| POBO.1b | 17 | 1 | 2 | 56 | 24 |  | 269 | | 83 |
| POBO.1c | 22 | 0 | 3 | 66 | 9 |  | 240 | | 78 |
| Mean | 25.33 | 0.33 | 1.67 | 56.00 | 16.67 |  | 238.33 | | 74.67 |
| SD | 10.41 | 0.58 | 1.53 | 10.00 | 7.51 |  | 31.53 | | 10.41 |
| POBO.2a | 50 | 0 | 0 | 18 | 32 |  | 182 | | 50 |
| POBO.2b | 46 | 0 | 0 | 25 | 29 |  | 191 | | 54 |
| POBO.2c | 37 | 0 | 3 | 43 | 17 |  | 203 | | 63 |
| Mean | 44.33 | 0.00 | 1.00 | 28.67 | 26.00 |  | 192.00 | | 55.67 |
| SD | 6.66 | 0.00 | 1.73 | 12.90 | 7.94 |  | 10.54 | | 6.66 |
| SM1.1a | 62 | 6 | 1 | 26 | 5 |  | 106 | | 38 |
| SM1.1b | 58 | 0 | 0 | 35 | 7 |  | 133 | | 42 |
| SM1.1c | 55 | 14 | 4 | 26 | 1 |  | 104 | | 45 |
| Mean | 58.33 | 6.67 | 1.67 | 29.00 | 4.33 |  | 114.33 | | 41.67 |
| SD | 3.51 | 7.02 | 2.08 | 5.20 | 3.06 |  | 16.20 | | 3.51 |
| SM1.2a | 28 | 0 | 2 | 44 | 26 |  | 240 | | 72 |
| SM1.2b | 21 | 0 | 1 | 40 | 38 |  | 274 | | 79 |
| SM1.2c | 28 | 0 | 3 | 33 | 36 |  | 249 | | 72 |
| Mean | 25.67 | 0.00 | 2.00 | 39.00 | 33.33 |  | 254.33 | | 74.33 |
| SD | 4.04 | 0.00 | 1.00 | 5.57 | 6.43 |  | 17.62 | | 4.04 |
| SM2.1a | 36 | 0 | 1 | 59 | 4 |  | 195 | | 64 |
| SM2.1b | 24 | 0 | 0 | 67 | 9 |  | 237 | | 76 |
| SM2.1c | 16 | 0 | 0 | 64 | 20 |  | 272 | | 84 |
| Mean | 25.33 | 0.00 | 0.33 | 63.33 | 11.00 |  | 234.67 | | 74.67 |
| SD | 10.07 | 0.00 | 0.58 | 4.04 | 8.19 |  | 38.55 | | 10.07 |
| SM2.2a | 26 | 1 | 3 | 41 | 29 |  | 246 | | 74 |
| SM2.2b | 22 | 2 | 7 | 41 | 28 |  | 251 | | 78 |
| SM2.2c | 31 | 0 | 5 | 39 | 25 |  | 227 | | 69 |
| Mean | 26.33 | 1.00 | 5.00 | 40.33 | 27.33 |  | 241.33 | | 73.67 |
| SD | 4.51 | 1.00 | 2.00 | 1.15 | 2.08 |  | 12.66 | | 4.51 |

Damage levels: zero (0) represents no genetic damage and 1 to 4 are the increasing levels of genetic damage. CO- (Negative Control). CO+ (Positive Control). NAT (Lettuces from Natuba); POBO (Lettuces from Pombos); SM1 (Lettuces from the Street Market - 1º selling point); SM2 (Lettuces from the Street Market - 2º selling point).

Table S3. Values of the chemical analysis of five soil samples obtained from Natuba cultivation area (NAT) through the Energy dispersive X-ray fluorescence (EDXRF), plus the central tendency values. Final values of central tendency are expressed in mg/kg.

| Soil samples (NAT) | | | | | | | | | | | | | | | |
| --- | --- | --- | --- | --- | --- | --- | --- | --- | --- | --- | --- | --- | --- | --- | --- |
|  | 1 | | 2 | | 3 | | 4 | | 5 | | Central Tendency | | | | |
| **Heavy metals** | **Mean** | **Uncertainty** | **Mean** | **Uncertainty** | **Mean** | **Uncertainty** | **Mean** | **Uncertainty** | **Mean** | **Uncertainty** | **Median** | **Max** | **Min** | **Mean** | **n** |
| Ni | 12,9 | - | 15,6 | 5,806863 | 17,6 | 6,139824 | 16,2 | 5,407371 | 18,3 | 6,006634 | 16,2 | 18,3 | 12,9 | 16,12 | 5 |
| Cu | 56,5 | 8,230354 | 74,3 | 8,526355 | 123,5 | 9,30047 | 79,9 | 8,626887 | 103,9 | 8,985473 | 79,9 | 123,5 | 56,5 | 87,62 | 5 |
| Zn | 149,1 | 21,76177 | 217,3 | 23,67646 | 291,6 | 26,34348 | 235,1 | 24,31692 | 269,6 | 25,3601 | 235,1 | 291,6 | 149,1 | 232,54 | 5 |
| Pb | 22,4 | - | 23,3 | - | 26,6 | - | 15 | - | 32,2 | - | 23,3 | 32,2 | 15 | 23,9 | 5 |

Ni: Nickel. Cu: Copper. Zn: Zinc. Pb: Lead. NAT: Natuba cultivation area. n: Number of samples.
